# The Placental Steroid Hypothesis of Human Brain Evolution

**DOI:** 10.1002/evan.70003

**Published:** 2025-06-20

**Authors:** Alex Tsompanidis, Graham J. Burton, Simon Baron‐Cohen, Robin I. M. Dunbar

**Affiliations:** ^1^ Autism Research Centre, Department of Psychiatry University of Cambridge Cambridge UK; ^2^ Loke Centre for Trophoblast Research, Department of Physiology, Development and Neuroscience University of Cambridge Cambridge UK; ^3^ Department of Experimental Psychology University of Oxford Oxford UK

## Abstract

The evolution of the human brain has long been framed in terms of sexual selection, with an emphasis on consistent but small on‐average volumetric differences between males and females. In this review, we present new molecular, genetic and clinical findings regarding neurodevelopment, cortical expansion and the production of sex steroid hormones, such as testosterone and oestradiol, by the placenta during pregnancy. We discuss converging evidence that on‐average sex differences are relevant for human evolution but are characterised by significant overlap between the sexes and more adaptations in female, rather than male, physiology. We also consider recent accounts and modelling of evolutionary pressures in large social groups, regarding competition and fertility. Finally, we bring these findings together and present a novel hypothesis for understanding human brain development and evolution, which emphasises the role of sex steroid hormones, their prenatal production by the placenta and their roles in regulating physiology, fertility and cognition.

## 1 Introduction

Sexual selection has long been thought to play an important role in human evolution [1]. Since Darwin first drew an analogy in the ‘Descent of Man’, between the brain and the peacock's tale, scientists have emphasised sex differences in human anatomy and aspects of cognition. However, comparative studies in nonhuman primates, as well as extinct hominin species, are now revealing that while these on‐average differences are consistent, they are relatively reduced in modern humans [2, 3]. Instead of overt displays of secondary sex characteristics, such as in the musculature of males, humans appear to have acquired more functional and anatomical adaptations in the reproductive system of females [4, 5, 6]. It remains unclear how sexual selection in humans may relate to the derived traits of the human brain, including its size, connectivity or emergent cognitive features such as sociality, memory or language. Recent advances in genomics, developmental neuroscience and molecular biology are providing novel insights into the potential biological systems that can link many of these human derived traits ‘in light of evolution’. The human placenta and particularly its synthesis of sex steroid hormones, may be an example of such a system.

## 2 Sex Differences and Human Anatomy

Human anatomy has been extensively compared to our species’ closest animal relatives, as well as extinct human species. Many human‐specific adaptations have thus been well described, including, but not limited to, a more gracile skeleton, retention of neotenous cranial features into adulthood, lack of terminal body hair [7], as well as more specific adaptations in the pelvis and the cranium [8, 9, 10]. Many of these anatomical features were adapted gradually, with some, such as the shape of the cranium, showing evidence of recent change, even after speciation [9]. When examining these features in a developmental framework, it becomes apparent that many of them interact significantly with aspects of development and particularly the mechanisms that contribute to sex differentiation [8], whether this is primary (i.e., during prenatal programming) or secondary (i.e., during puberty and throughout life).

**Figure 1:**
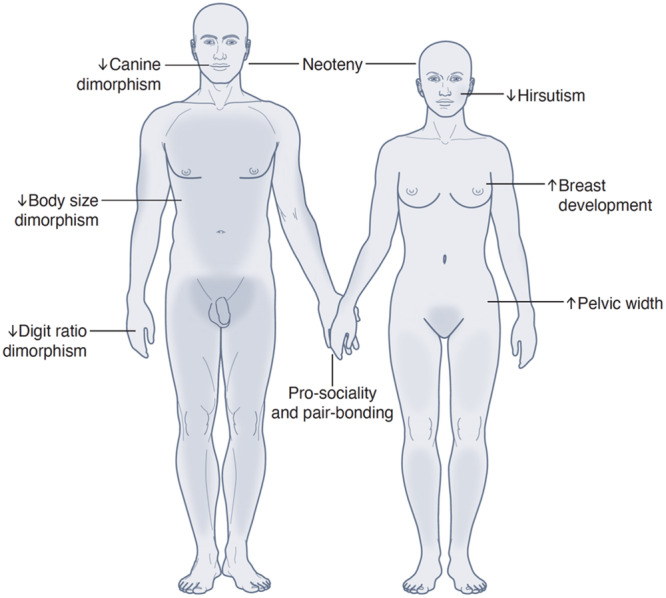
Sex differences in anatomically modern, adult humans are characterised by reduced male‐type dimorphism and increased female‐type dimorphism, compared to other hominids.

Comparative analyses of adult body weight, muscle mass, canine tooth length and other features, all show reduced sex differences in humans, compared to most Old‐World primates (Figure 1) [3]. These anatomical features are often attributed to the effects of testosterone and male‐specific pressures to compete for access to reproduction and resources. This trend towards uniformity of the sexes is mirrored in human adaptations in male‐driven competition and aggression, which is deemed less reactive compared to other primates, such as chimpanzees [11, 12].

On the other hand, human females show more adaptations in their sex‐specific physiology and behaviour. Compared to most primates that exhibit transient changes in physiology that correspond to fertility, human females develop more permanent secondary characteristics that signal postpubertal development, independently of menstrual fluctuations [13]. The main example, human breast development, can be directly attributed to oestrogen exposure, as shown extensively in the preponderance of hormone‐dependent breast tumours (the most common form of cancer in human females) [14], rare conditions that feature gynaecomastia in males (e.g. aromatase excess) [15], as well as genome‐wide‐association studies of the trait [16].

In addition, the menstrual cycle in humans is slightly accelerated compared to Old World primates, with an average length of 28 days (compared to 33‐35 days in Old World primates). The regulation of cycle length depends on the effects of circulating hormones and their signals in the hypothalamus and pituitary; the two brain regions that are responsible for regulating endocrine homoeostasis and the function of the gonads (i.e., the hypothalamus‐pituitary‐gonadal axis, ‘HPG’). These signals can be inhibitory, excitatory or switch between the two, depending on the timing of the cycle (e.g., the level of maturation of the follicles and of the uterine lining). Oestradiol, one of the most potent oestrogens, is a characteristic example of this property, as it can switch its effects on the HPG axis from inhibition to excitation, as its levels gradually increase during the maturation of a follicle [17]. This leads to a rapid change in the HPG axis that eventually results in ovulation. Therefore, sensitivity to oestradiol is crucial for the regulation of the timing of the cycle.

Finally, most human females characteristically have a wider pelvis, compared to males, from puberty onwards. Contrary to other anatomical features, the extent of sex differences in the pelvis appears to have increased throughout the *Homo* lineage, with speculation that this was in response to early pressures from bipedalism (in both sexes), as well as the later need (in females) to facilitate the delivery of fetuses with larger crania [10]. Interestingly, recent evidence from human pelvic growth trajectories, rare genetic syndromes, as well as studies in other primate species and rodents, show that pelvic growth can be attributed to oestrogens, rather than growth hormones [18] and that its rapid evolutionary change is associated with “changes of degree” in human endocrinology [19], rather than a specific genetic adaptation.

Taken together, these observations indicate that oestrogenic effects are particularly pronounced, relative to androgens, when considering sex differentiation during human development, as well as in the context of evolution. This is consistent with comparative findings on the 2D:4D digit ratio, which is thought to reflect the balance between oestrogens and androgens *in utero*. This ratio has been found to be particularly high in humans, compared to both anthropoid primates, as well as other extinct species in the *Homo* lineage [20].

***Note on neoteny**:* Similar to the rest of the skeleton, certain anatomical features on the face (e.g., brow ridge thickness, facial length) appear to have undergone gradual evolutionary changes in the genus *Homo*, resulting in a pattern that matches a juvenile phenotype for other hominids. Interestingly, this gradual trend towards “neoteny” or “paedomorphosis” has also been described as “craniofacial feminisation”, given the apparent overlap of these features with baseline sex differences in many populations [8]. Clinical studies in modern humans also show that facial morphology and skeletal maturation are significantly affected by sex steroid hormone levels, such as oestradiol, particularly during prenatal life [21, 22]. More research is needed to test if there is an overlap between human neoteny and mechanisms of sex differentiation (including steroid receptor resistance), or if both can be attributed to other processes related to domestication, such as the neural crest hypothesis [23].

Developmentally, features such as sex differentiation, digit ratios and steroid sensitivity are all thought to be shaped by exposure to steroid hormones during early life and particularly in the womb. Many lines of evidence in humans, as well as animal models, have shown that prenatal steroid hormones, can have long‐term ‘organisational’ effects on the brain of the developing foetus. These prenatal effects set the stage for the ‘activational’ effects of hormones during and after puberty, during which an individual's gonads and adrenals start producing sex steroids again, bringing about fertility and the development of secondary sexual characteristics that signal maturity. However, during prenatal life, sex steroid levels are not regulated directly by the gonads but rather by the placenta, a tissue of crucial developmental significance for the health of pregnancy, the supply of nutrients and for the development of the brain.

## 3 Placental Function and Human Evolution

The shape, size and morphology of the placenta are important factors in the context of evolution, as they exhibit considerable variability, are often species‐specific and are quick to adapt to external pressures to secure both short‐term and long‐term viability [24, 25, 26]. The predominant functions of the placenta are to regulate the transfer of nutrients and oxygen from the mother to the foetus, and to orchestrate maternal adaptations to pregnancy. In humans, this ‘maternal investment’ has long been proposed to be particularly relevant for the development of large brains and this is now confirmed by modelling [6, 27]. Nevertheless, there appears to be a complex relationship between placental traits, such as the degree of invasiveness, of maternal‐foetal interdigitation, and brain size, as these traits appear to contribute more directly towards gestational length than directly to the degree encephalisation across species [28]. Therefore, placental morphology may be the target of more complex evolutionary dynamics that are species‐specific and feature trade‐offs between maternal and offspring interests.

In humans, these evolutionary dynamics may significantly interact with sex differences. Recent findings from genomics, clinical epidemiology and molecular biology, all confirm that the human placenta is significantly affected by biological sex. Sex differences in placental gene expression start at conception and are mainly attributed to X‐chromosome dosage [29, 30]. Proteins involved in placental angiogenesis also differ by sex as early as the first trimester [31, 32]. Placental production of sex steroid hormones is also affected by the sex of the foetus, with consistent sex differences in their levels [33, 34]. Pregnancies carrying male fetuses are also more likely to fail in the first 3 months after conception, as well as exhibit gestational hypertension, spontaneous preterm birth and reduced ability to adapt in the face of prenatal adversity [35, 36]. In the context of evolution, these sex differences in adaptation and viability have been attributed to altered ‘strategies’ for each sex when it comes to energy usage, particularly in the presence of maternal adversity, with males “prioritising” nutrient uptake, compared to more adaptive changes in the placentas of females [35, 37].

**Figure 2:**
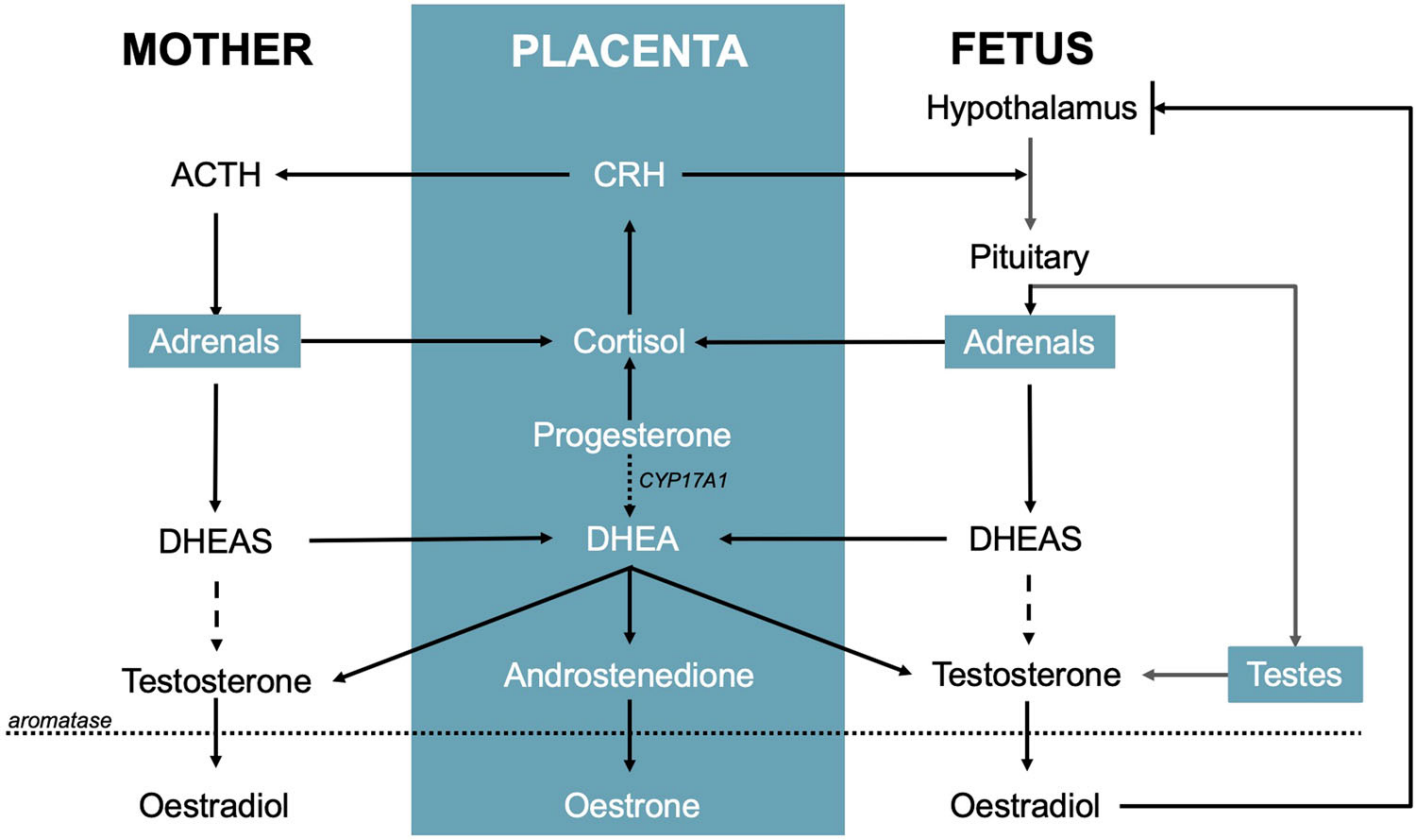
Aspect of steroidogenesis in the maternal‐placental‐foetal interface. Males differ to females due to the activation of the foetal testes, leading to small, baseline sex differences in prenatal androgens.

Among mammals, anthropoid primates are distinctive in their retention of a haemochorial placenta [38]. In this configuration, placental trophoblast cells penetrate deeply into the endometrial tissue, progressively modifying maternal vasculature throughout gestation. This structural arrangement facilitates direct endocrine communication, enabling both placental steroid synthesis and effective regulation of the integrated maternal‐placental‐foetal system (Figure 2).

Primate placentation is distinguished by its ability to synthesize chorionic gonadotropin (hCG) during early gestation, derived from a duplication of the luteinizing hormone beta subunit gene. Interestingly, humans exhibit additional gonadotropin gene duplications, resulting in more functional hCG variants than other primates [39]. hCG orchestrates early placentation and maintains the corpus luteum, peaking in late first trimester before initiating placental steroidogenesis, which is subsequently maintained by placental progesterone. Placental progesterone serves as a substrate for DHEAS production in both maternal and foetal adrenal glands. Through haemochorial transport, DHEAS then returns to the placenta where it is converted to androgens (specifically androstenedione and testosterone), which subsequently undergo rapid aromatization to form oestrogens (oestrone and oestradiol). As pregnancy progresses into its latter half, the placenta achieves adequate expression of CYP17A1, further amplifying placental sex steroid hormone production through to delivery [40, 41]. Another primate‐specific adaptation, placental CRH, regulates both the maternal and foetal hypothalamic, creating positive feedback loops that progressively elevate sex steroid levels to unprecedented concentrations [42] (Figure 2).

This hormonal elevation occurs without proportional increases in steroid‐binding proteins, particularly alpha‐fetoprotein (AFP), which declines in foetal circulation during late pregnancy [43]. Primates show distinct AFP gene regulation, with human‐specific intronic repeat elements being absent in other great apes, though the functional significance of this variants remains unclear [44, 45].

The few comparative studies between humans and other apes demonstrate enhanced prenatal steroidogenesis in our species, with substantially higher gestational oestrogen peaks [46, 47] and elevated placental aromatase expression compared to macaques [48]. These findings suggest that the primate placenta has evolved into a specialized steroidogenic hub, with particularly pronounced adaptation in humans, resulting in exceptionally high prenatal oestrogen levels.

***Note on the placenta and transgenerational transmission***: Placental steroidogenesis links the maternal and foetal HPA/G axes [49], with potential developmental effects across generations. According to this notion, placental steroids can ‘condition’ the responsiveness and sensitivity of the hypothalamus of the next generation. When the female offspring become pregnant themselves, they utilise their ‘conditioned’ HPA/G axis in a new cycle of steroid production and foetal neurodevelopment of their own offspring. This ‘transgenerational transmission’ has been shown with specific gene expression networks in the hypothalamus, as well as for the first time in humans, in cases of polycystic ovary syndrome (PCOS) [50]. In this complex but relatively common endocrine syndrome, women with the condition generally have elevated levels of androgens, which during pregnancy further ‘condition’ the HPA/G axis of their daughters, who in turn are more likely to have PCOS and hyperandrogenaemia during their life, thus propagating these endocrine traits in a transgenerational manner across the female line. The evolutionary significance of these effects, particularly with regard to pregnancy or brain development, has been underexplored in humans.

The main difference between males and females during prenatal life is the presence of the foetal testes in males, which briefly produce androgens in the early second trimester, termed the prenatal masculinisation window (PMW). This brief surge is thought to masculinise the external genitalia but has not been conclusively shown to affect the brain or behaviour in humans [51]. Males also appear to have higher levels of placental DHEA than females [30], which is one of the main sex steroid precursors that give rise to both androgens and oestrogens. Nevertheless, males also have higher levels of placental aromatase than females [52, 53], arguably leading to the prompt conversion of maternal and gonadal androgens to oestrogens, potentially reducing androgenic effects and the ratio of androgens to oestrogens.

While rodents rely to a large extent on oestrogens formed from androgens for their masculinisation, in humans signalling downstream of the androgen receptor appears to be sufficient for masculinisation of anatomy, as evidenced by rare cases of 5a reductase deficiency (which prevents the synthesis of the potent androgen receptor ligand DHT), congenital complete androgen insensitivity (where XY individuals have external genitalia that are phenotypically female) and even more rare forms of aromatase deficiency (where XY individuals are phenotypically male) [54, 55]. Therefore, it is intriguing to consider the developmental significance of placental aromatisation and oestrogens in particular, since these do not contribute to ‘masculinisation’ in primates, but show evidence of evolutionary adaptations that favour a more general steroidogenic excess during prenatal development.

## 4 Placental Sex Steroids and the Brain

Recent experiments involving human iPSC‐derived brain organoids have also shown that androgens can directly regulate cortical expansion [56]. Specifically, administering testosterone, or its potent derivative DHT, has been shown to increase the proliferation of cortical progenitors, increasing the overall neurogenic pool, during a critical time‐point in the formation of the cortex. This is in agreement with small but consistent differences between the sexes at birth, with human male neonates averaging 3.5% more whole brain volume than females, even when controlling for differences in body size or weight [57, 58].

Compared to their androgen precursors, oestrogens appear to have a more multi‐faceted and context‐specific role in human brain development. *In vitro* assays in iPSC‐derived human neurons, indicate the oestrogen signalling is important for the formation and stabilisation of complex synapses [59, 60, 61, 62, 63]. Animal models also show that oestradiol, the most potent of the oestrogens, can increase the number of spines and the stellation of neighbouring astrocytes [64, 65]. Increased spine number and glial cell specialisation have recently been shown to be a characteristic feature of human neurons compared to other primates [66, 67, 68]. Oestrogens can also induce the expression of the brain‐derived neurotrophic factor (BNDF) gene [69, 70, 71, 72], which in turn is associated to a human‐accelerated‐region (HAR) [73]. In addition, oestrogens can interact and upregulate Notch signalling in humans, as has been shown not only in cellular studies of endothelial or cancer lines [74, 75, 76, 77], but also in the context of neurodevelopment based on findings in rodents [78]. The Notch pathway has also been shown to have acquired human‐specific modulators, such as NOTCH2NL, which regulate radial glial biology, contribute to cortical expansion and, in the presence of mutations or disruptive copy number variants, have been associated with microcephaly and autism [79, 80]. Finally, most of the recently identified microcephaly genes have acquired partial oestrogen‐response‐elements, which co‐evolved with other brain size genes in primates [81]. In experimental assays, oestradiol appeared to downregulate these genes, with the study's authors suggesting that this may contribute to reduced relative brain size in human females, compared to males. However, during prenatal development, there is no evidence of sex differences in oestradiol levels or of an oestrogen‐mediated “feminisation” programme in humans. It is therefore more likely that in humans, aromatisation of testosterone to oestradiol in the brain, may act as a rate‐limiting mechanism in both males and females, balancing androgen‐mediated neuronal proliferation with oestrogen‐regulated synapse formation and connectivity. Aromatase would be particularly important in mediating this ‘trade‐off’ and, accordingly, has been shown to be expressed widely in the human brain during prenatal development [52, 53].

Particularly for aromatase, primates appear to have acquired redundancies, in the form of multiple independent promoters that induce its expression. These exceed the number of promoters found in rodents [14], are tissue‐specific and are thought to maintain high levels of aromatase expression specifically in the brain and in the placenta [14].

## 5 Placental Sex Steroids and Human Cognition

The evolutionary significance of placental sex steroid hormones is further indicated by their association to many aspects of human cognition and behaviour. Accumulating evidence from studies in developmental neuroscience and behavioural psychology suggest that prenatal sex steroid levels, in both males and females, are associated with the development of many human cognitive skills, such as vocabulary acquisition [82], theory of mind and the matching of emotional states to facial expression [83]. This is reflected in small but consistent sex differences in the cognitive trajectories of girls and boys, with the latter being more likely to show delays in acquiring these features of social cognition and to be diagnosed with conditions such as autism, particularly in childhood [84, 85].

In terms of normative development, sex differences in the levels of prenatal steroids, as well as of the placental growth factor (PLGF), have been associated with autistic traits in the offspring [86, 87, 88]. Placental genomic profiles can also predict a series of cognitive and behavioural traits in the offspring, related to attention, sensory integration or later IQ [89, 90, 91]. The presence of a developmental ‘placenta‐brain’ axis is not a uniquely human feature. In rodents, placental allopregnanolone (a steroid byproduct in the androgen ‘backdoor’ pathway) has been shown to reach the developing cerebellum and shape the postnatal social behaviour of the males [92].

These associations indicate that prenatal steroids and their production by the placenta contribute to neurodevelopment and the variance in many cognitive traits that are pronounced in humans, such as pro‐sociality, theory‐of‐mind and analytical thinking. The co‐evolution of these traits in humans may be understood in the context of the ‘social brain’ hypothesis.

## 6 Sex Differences and the Social Brain

The social brain hypothesis [93, 94] provides a framework to explain the link between two traits that can be inferred from the palaeoanthropological record, namely group size and cortex size. According to this theory, the need to maintain larger groups arguably led to adaptations in both physiology and psychology, which enabled a new system of managing both polygyny and polyandry [11, 95]. It has long been proposed that pair‐bonding behaviours in humans, albeit intermittent, emerged as an effective strategy to address these pressures (i.e. ‘the hired gun’ hypothesis) [96]. Yet, after many years of multi‐disciplinary research, other types of social relationships, such as friendships and alloparenting are now increasingly recognised as important behavioural adaptations that can also facilitate social cohesion in large group sizes [5, 97, 98]. In addition, population research now shows that pair‐bonding behaviour in humans is more complex and shows important differences compared to other primate species that practice it, such as gibbons and small cebids [99]. For example, there are consistent sex differences in the seeking, maintenance and experience of pair‐bonds. Although there is considerable overlap between the sexes (as with most cognitive traits) [2], in humans pair‐bonds appear to be ‘enforced’ more effectively and frequently on‐average by females, rather than by males [4, 99, 100, 101].

**Note on variance and gender**: It should be noted that while sex differences in reproductive and social behaviour have been consistently reported in human populations [102], they are often modest, highly context‐dependent, and vary across populations. Compared to physical traits such as height or postpubertal body proportions, behavioural differences between the sexes tend to have smaller effect sizes and exhibit substantial overlap, supporting the Gender Similarities Hypothesis [103]. Moreover, there is considerable individual variation within each sex and even across different behavioural domains within the same individual. In addition, as first noted by Darwin, variance in human males may be somewhat greater, compared to human females, for some cognitive traits, as well as physical features, including brain structure (i.e. the ‘variability’ hypothesis) [104, 105]. However, sex differences in variance are also small, and their evolutionary relevance remains uncertain, particularly since they do not consistently align across physical and social traits in other species [106]. These observations may still align with the hypothesis presented here, which highlights the apparent evolutionary reduction of sex differences in humans, as well as the effects of sex steroid hormones on the brain. After all, sex steroid hormones are highly dynamic and fluctuate greatly within and between individuals. Notably in humans, male sex differentiation depends on the cumulative effects of both prenatal and pubertal steroid influences, whereas female differentiation is thought to be shaped chiefly by postnatal processes that add on a prenatal ‘default’. These biological patterns may contribute to slightly greater variance in males, but without undermining the importance of sex steroid hormones, or the broader pattern of convergence in physiology and cognition between the sexes.

Population modelling studies now indicate that minimising aggression in males and maintaining high rates of fertility in females are both important for the maintenance of large social groups. Studies in mammals show that these are connected, as increases in group sizes are associated with increases in male competition, which in turn lead to a reduction in female effective fertility [4]. This ‘infertility trap’ can be due to overt acts of aggression, such as infanticide [107] but also due to increasing stress in females due to coercion, harassment and disruption of child‐rearing [4]. A recent 'evo‐devo' model across seven hominins also identified the energy costs associated with fertility (specifically follicular count in females) as a significant constraint on the evolution of the human brain [108]. Follicular count is a trait that is also heavily regulated by oestrogenic signalling in humans [109]. Interestingly, in addition to their effects on neural connectivity, oestrogens also regulate the oxytocin system, resulting in upregulation of the gene and of its receptor in both animal models and humans [110, 111, 112].

**Figure 3:**
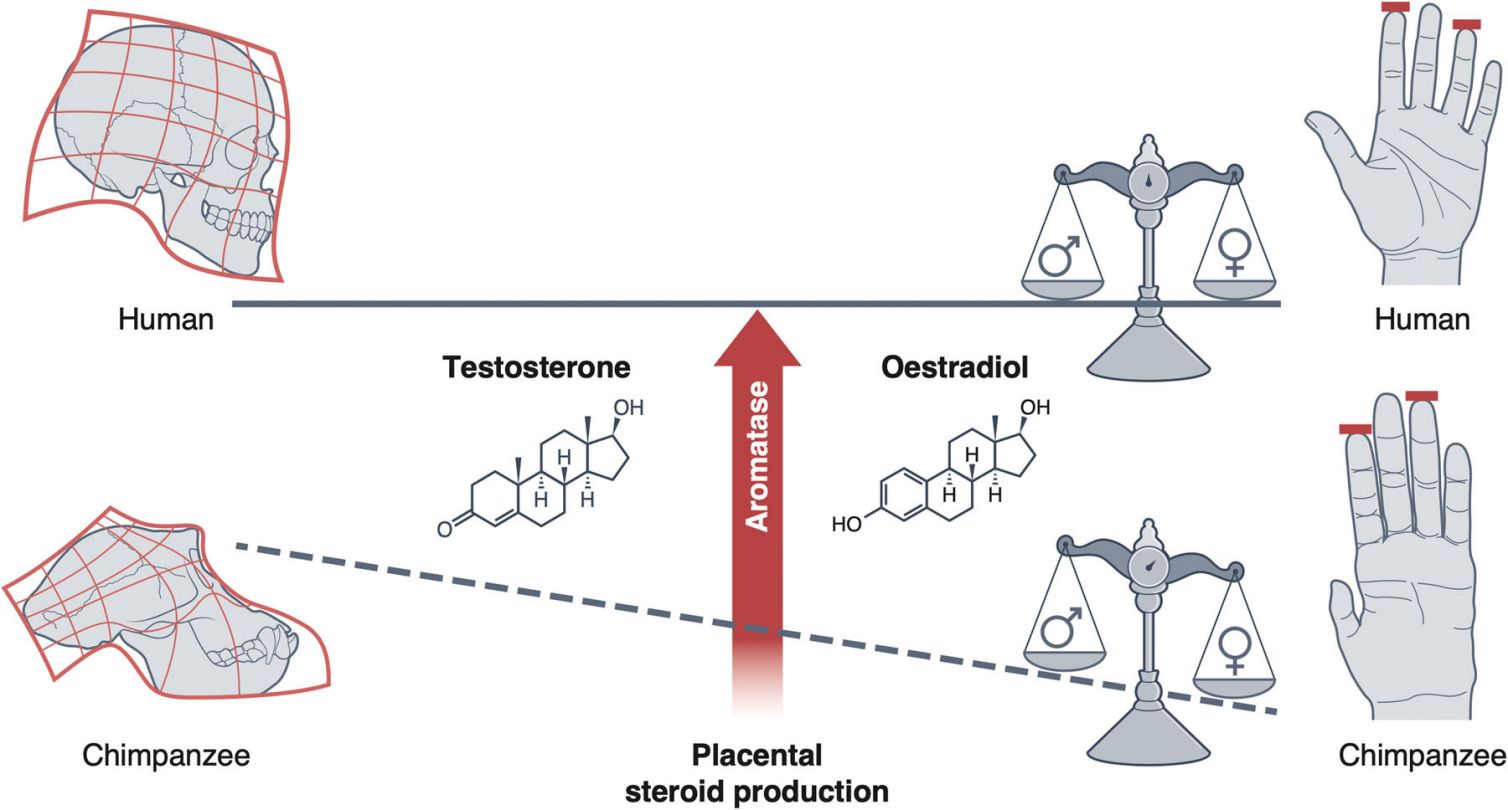
A high rate of aromatisation in the placenta, may have led to a reduction in male‐type sex differences in humans (e.g., in digit ratios), compared to other hominids, while maintaining higher levels of steroidogenesis.

Since oestrogens are formed from androgens, a hypothetical adaptation to increase oestrogen levels could also lead to high levels of androgens. Developmentally, it has been proposed that the ratio of testosterone to oestradiol, rather than testosterone levels alone, are a better proxy for ‘masculinising’ anatomy, such as digit ratios [113, 114]. Consequently, high levels of sex steroids, including testosterone, may not necessarily be linked to high levels of masculinisation, provided that aromatisation is efficient in the target tissues and prenatally in the placenta (Figure 3). Nevertheless, elevated steroidogenesis, particularly during prenatal life, could still potentially be linked to ‘trophic’ effects on brain volume and neuronal connectivity, in light of recent evidence from molecular studies outlined above [56, 59, 60, 61, 62, 63].

Steroidogenic excess, coupled with reduced androgenic effects, may not be a feature that is unique in human evolution. New World monkeys such as marmosets and tamarins have very high levels of circulating sex steroid hormones, including testosterone [115], but appear to be spared of androgenic increases in male‐to‐male aggression. In fact, they have been reported to have complex sociality and varying degrees of pair‐bonding [4, 116]. Their digit ratios are similar to humans’, albeit more variable [20]. The precise mechanism for this steroidogenic excess is unknown, but it has been proposed to involve a form of steroid resistance that may be countering inhibitory feedback [115]. This excess appears to have resulted in a new homoeostatic state that is more oestrogenic than androgenic, which may in turn facilitate more social bonding than male competition. Similar adaptations may therefore account for these traits in humans.

## 7 The Co‐Evolution of the Human Brain With Adaptations in Placental Function and Steroidogenesis

**Figure 4:**
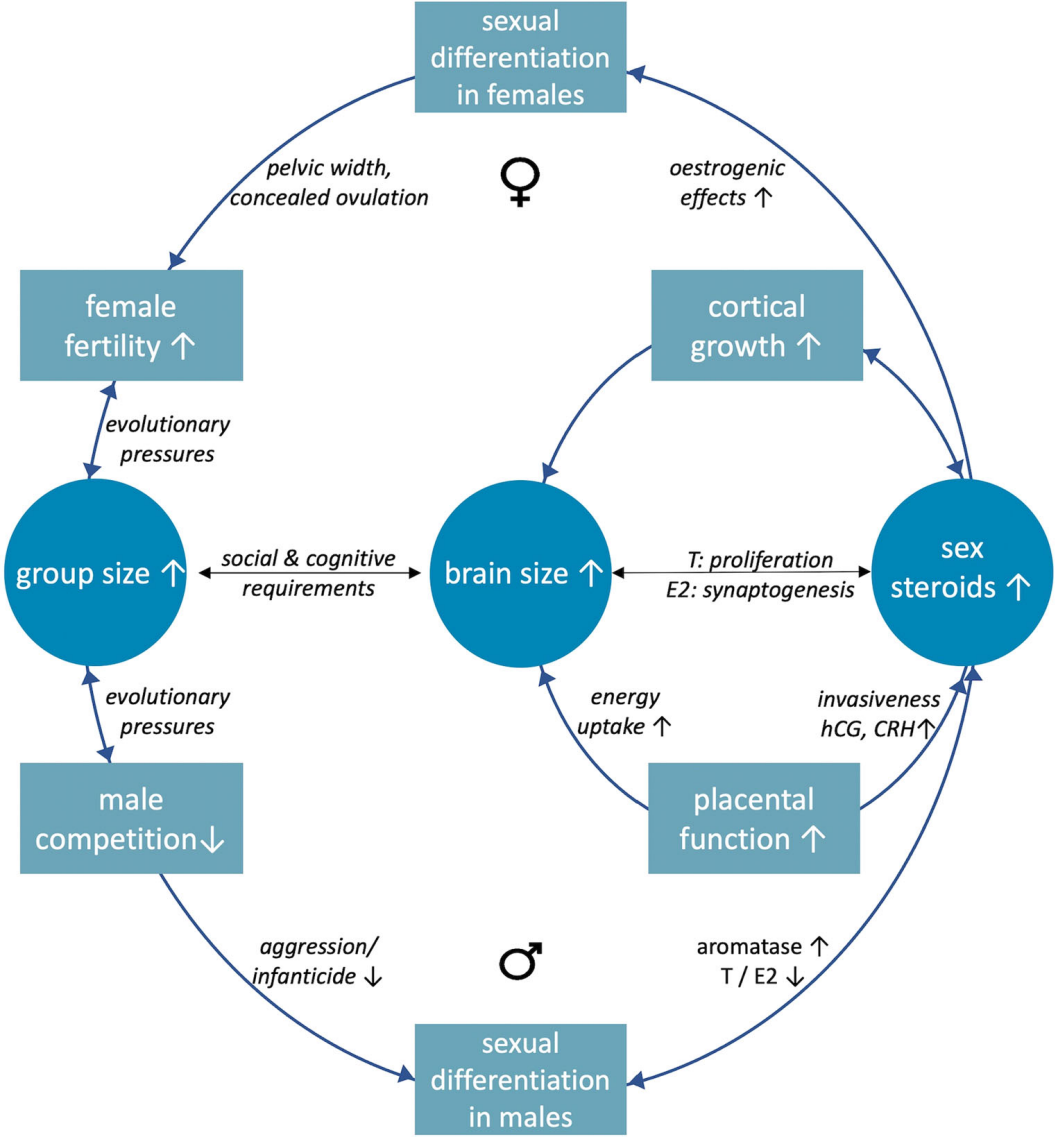
Circular diagrams of the co‐evolution of related traits in humans. Outer circle describes the ecological pressures stemming from the need to maintain large group sizes [4, 11]. Inner circle on the right shows the developmental link between brain growth and placental function, with a particular emphasis on the effects of placental sex steroids on neurodevelopment, based on recent findings [28, 56, 63]. T: testosterone, E2: oestradiol, hCG: human choriogonadotropin, CRH: placental corticotropin. Summary of the hypothesis: pressures to reduce infanticide, male aggression and to ensure female fertility in larger groups, may be linked to adaptations in placental function and steroidogenesis, which in turn resulted in increases in brain size and connectivity in modern humans.

The findings outlined above, may be organised in the following framework of understanding for human brain evolution (Figure 4).

In summary, based on evolutionary pressures to:

a. increase their group size [93],
b. maintain low levels of male‐to‐male, intergroup aggression and low levels of infanticide [4],
c. as well as high rates of female fertility [4] and
d. effective social relationships that include friendships, pair bonds and alloparenting [5, 11, 97]

Human*s* evolved adaptations for:

a. increasing prenatal steroids via the maternal‐placental‐foetal interface [46, 47].
b. while maintaining high levels of aromatisation in the placenta and the brain [14, 52, 53].

These prenatal phenomena resulted in the gradual co‐evolution of the following traits in hominins leading over time to the speciation of *Homo sapiens*:

a. an increase in the number of neural precursors in the cortex due to androgenic excess, contributing to an increase in brain size [56].
b. an increase in functional connectivity in the cortex and cerebellum due to oestrogenic excess [59, 60, 63].
c. A developmental modulation of the oxytocin network due to steroidogenic effects on the expression of the oxytocin gene and distribution of its receptor [110, 112, 117].
d. Other developmental effects influencing puberty and reproductive endocrinology, such as persistent sexual characteristics that conceal ovulation in females [13].
e. Reductions in postpubertal male‐type shifts in musculature, facial morphology and canine tooth length [2, 3, 8], and finally.
f. a reduction in reactive aggression between males [12], that led in turn to a reduction in infanticide and the maintenance of high rates of fertility in large social groups.

These adaptations may have been the result of cumulative transgenerational effects that are similar to the reported propagation of hyperandrogenaemia in the female line, leading to increased steroidogenesis with each generation [50]. Interestingly, this process may not have required the emergence and fixation of additional genetic variants, relying instead on relatively quick (in evolutionary time) ‘changes of degree’ in the endocrine systems already present in the primate line, as has been proposed before for the human pelvis [19]. Mate selection, particularly by females, based on associated behavioural characteristics, could have further accelerated this rate of adaptation. Therefore, the framework outlined here could be applied across the evolutionary history of *Homo sapiens*, and particularly when pressures on group size and social cohesion were present. Evidence from the fossil record indicates gradual continuing changes in facial morphology, digit ratios [8], as well as the shape of the cranium [9], which may be consistent with this notion.

***Note on the importance of the matriline***: This account is consistent with emerging evidence of the importance of the matriline in human evolution. Genetic variance analyses now indicate that females contribute as much [118] or even more [119] to the diversity of the species than males. Behaviourally, females are also on‐average more selective with mates than are males, across human populations [99]. Cortical genes also show more maternal than paternal imprinting [120]. The evolutionary interests of the matriline may also be evident in other developmental differences, such as a female advantage in longevity [121], and a so‐called “female protective effect” for neurodevelopmental conditions such as autism [122, 123].

## 8 Future Research Directions

More research is required to confirm if transgenerational transmission extends from traits such as hyperandrogenaemia in females, to aspects of placental function and/or the anatomical or neurodevelopmental features highlighted above. Future genetics studies could also test for signatures of recent, human‐specific adaptations in gene networks associated with sex steroid sensitivity, placental function and pro‐sociality. In terms of human development, more work is needed to elucidate how variance in placental steroid levels and placental aromatase activity can be linked to sex differentiation (particularly during puberty) or to specific aspects of social brain development and social cognition. Finally, in terms of phylogeny, comparative studies between humans and nonhuman primates are warranted, to replicate previous work on steroid levels [46, 47] and to further understand how the placenta may adapt to species‐level pressures related to group size and sexual selection.

## 9 Summary and Conclusion

In the *Descent of Man*, Charles Darwin was the first to speculate that the human brain evolved under pressures linked to male competition and reproductive success. While on‐average anatomical and behavioural sex differences in human societies are evident and consistent, their overall extent for human evolution may need to be reconsidered. Anatomical sex differences are relatively reduced in human males, while male behaviour is less hostile to other males and their offspring, compared to other hominids. Recent discoveries in developmental psychology and neuroscience indicate that all sex steroid hormones, including oestrogens, may be elevated prenatally in humans, compared to other primates, and that they can contribute to brain growth, neuronal connectivity and the development of social cognition. It is then intriguing to consider that human speciation involved adaptions in the physiology of pregnancy and the placenta, which resulted in changes in the human HPG/*A axis* and, in turn, modulated reproductive behaviour, increased the size and connectivity of the human cortex and ultimately expanded the socio‐cognitive capacities of all humans regardless of their sex.

## Conflicts of Interest

The authors declare no conflicts of interest.

## Data Availability

No original data were generated for the purposes of this study.
